# Amyloidosis in Alzheimer’s Disease: Pathogeny, Etiology, and Related Therapeutic Directions

**DOI:** 10.3390/molecules27041210

**Published:** 2022-02-11

**Authors:** Chen Ma, Fenfang Hong, Shulong Yang

**Affiliations:** 1 Experimental Center of Pathogen Biology, Nanchang University, Nanchang 330006, China; machen1008@163.com; 2 Queen Marry College, School of Medicine, Nanchang University, Nanchang 330036, China; 3 Department of Physiology, College of Medicine, Nanchang University, Nanchang 330006, China; 4 Department of Physiology, Fuzhou Medical College, Nanchang University, Nanchang 344099, China

**Keywords:** Alzheimer’s disease, amyloidosis, amyloid beta protein, amyloid precursor protein

## Abstract

The amyloid hypothesis of Alzheimer’s disease has long been the predominant theory, suggesting that Alzheimer’s disease is caused by the accumulation of amyloid beta protein (Aβ) in the brain, leading to neuronal toxicity in the central nervous system (CNS). Because of breakthroughs in molecular medicine, the amyloid pathway is thought to be central to the pathophysiology of Alzheimer’s disease (AD). Currently, it is believed that altered biochemistry of the Aβ cycle remains a central biological feature of AD and is a promising target for treatment. This review provides an overview of the process of amyloid formation, explaining the transition from amyloid precursor protein to amyloid beta protein. Moreover, we also reveal the relationship between autophagy, cerebral blood flow, ACHE, expression of LRP1, and amyloidosis. In addition, we discuss the detailed pathogenesis of amyloidosis, including oxidative damage, tau protein, NFTs, and neuronal damage. Finally, we list some ways to treat AD in terms of decreasing the accumulation of Aβ in the brain.

## 1. Introduction

Alzheimer’s disease (AD) is a late-stage neurodegenerative disorder associated with advanced progressive dementia [1]. It ranks the fifth leading cause of death worldwide, affecting approximately 45 million people [2]. The symptoms of AD include emotional fluctuation, sleep disorders, behavior changes, and cognitive decline [3,4]. When AD progresses to advanced stages, it can cause severe symptoms such as malnutrition, multi-organ failure due to neuronal necrosis, and even brain death [5]. According to a 2019 report, more than 47.7 million people worldwide have Alzheimer’s disease, the incidence of which is rising sharply every year. If this trend continues, Alzheimer’s will affect 115 million people worldwide by 2050 [6]. The death toll from Alzheimer’s disease is likely to exceed the number of deaths recorded to be caused by it on death certificates [7]. Despite many basic studies on the pathogenesis of Alzheimer’s disease, the potential for promising findings being translated into clinical treatments remains limited.

After years of research, scientists initially considered that Alzheimer’s disease is a complex disease with genetic and environmental factors and other complex causes, such as gender, age, family history, and Down syndrome [8,9,10]. However, the actual pathogenesis of Alzheimer’s disease is still unclear. The amyloid cascade hypothesis proposed by scientists has become the primary model of AD pathogenesis [11].

According to the hypothesis, the accumulation of toxic amyloid beta-protein (Aβ) in the central nervous system (CNS) is the main cause of Alzheimer’s disease [12]. Extracellular abnormalities of Aβ levels in the brain may lead to the accumulation of Aβ, forming a structure rich in β-Sheet. After forming oligomers, they recombine into fibrils to form amyloid plaques. Moreover, the aggregation of oligomers is harmful to cells. In the brains of AD patients, oligomers penetrate the cell membrane, triggering a series of pathological events and ultimately leading to cell dysfunction and death [10,13]. Therefore, a clearer understanding of how amyloid accumulates in the brain of AD patients is important for both the development of more effective treatments and the prevention of AD. At the same time, both excessive amyloid production and impaired clearance may lead to accumulation in the brain. This article will describe the pathogenesis of amyloidosis in AD and some possible targets for the treatment of AD.

## 2. Role of Amyloidosis and Its Precursor in AD

### 2.1. Amyloidosis

Amyloidosis is a clinical and pathological condition in which amyloid accumulates in various organs and cells of the body, forming amyloid plaques for complex reasons, leading to organ dysfunction. It can be hereditary or acquired [14]. Depending on the location of amyloid fibers’ deposition, amyloidosis is divided into two groups, one is localized amyloidosis, that occurs in a specific area of a single tissue, and the other is systemic amyloidosis, which occurs throughout the body [10,15]. Amyloid plaques are made up of amyloid proteins [16]. Amyloid β peptide (Aβ) is the main component that plays a significant role in the pathogenesis of AD and is considered to be the leading cause of AD development [14,17,18]. In addition to neurofibrillary tangles (NFTs)—which are caused by microtubule-related proteins, excessive phosphorylation of tau proteins, and neuronal loss—amyloid plaques are also the hallmark of AD.

### 2.2. Amyloid Precursor Protein

Amyloid precursor protein (APP) is a single-pass transmembrane protein concentrated in neuronal synapses, which is highly expressed in the brain [17,18,19]. Compared to Aβ, APP is a larger precursor molecule that is generated by brain neurons, blood vessels, blood cells, and a small number of astrocytes. Subsequently, APP is cleaved by two hydrolyses, including β-secretase extracellular and γ-secretase intracellular, to generate Aβ [20]. Although the physiological functions of APP remain unclear, it plays an essential role in brain development, memory, and synaptic plasticity [21,22]. It not only can protect the neurons but also modulates intercellular interactions, regulating the growth of neurons and synaptic plasticity [23]. Mutations in APP have been shown to lead to an increase the synthesis of Aβ, which form senile plaques and cause degenerative changes in peripheral neurons [24]. Mutations in the transmembrane region of the amyloid precursor protein have been reported to alter the ratio of residue 42 of Aβ protein (Aβ42) and residue of 40 Aβ protein (Aβ40), particularly in the early onset familial Alzheimer’s disease [25].

### 2.3. Amyloid Beta Protein

Amyloid beta protein (Aβ) is a short 4.2 kDa peptide consisting of 40–42 amino acids, The precursor of Aβ is amyloid precursor protein (APP). On the cell membrane of brain neurons, APP is cleaved in the presence of secretase to produce Aβ peptides [25]. Amyloid proteins are misfolded proteins that display a stable secondary structure. Moreover, abnormal accumulation of Aβ leads to neurotoxicity in the brain [14]. Monomeric Aβ fragments are soluble substances. They aggregate into insoluble oligomers, subsequently forming neurological plaques. Our body has several mechanisms to expel Aβ from the brain, including the transfer through the receptor for advanced glycosylation end products (RAGE) and density lipoprotein receptor-related protein-1 (LRP1) [26,27,28]. Experimental studies have shown that an imbalance between Aβ synthesis and clearance leads to problematic metabolism, which results in protein misfolding, aggregation, and extracellular accumulation, eventually forming amyloid plaques [29,30].

**Figure 1:**
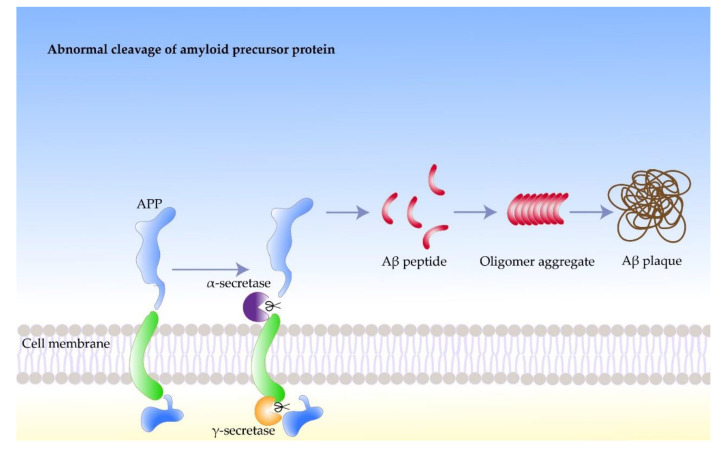
The formation process of amyloid beta fibrils.

Nerve cells produce more Aβ than other cell types, which play important roles in normal physiological activities of the central nervous system, such as intercellular signaling [31]. Aβ was found to accumulate in both traumatic brain injury patients and Parkinson’s patients, suggesting an association of amyloid with neurodegenerative diseases [32]. The response of the body to chronic stress increases the rate of neuronal protein synthesis, which leads to the accumulation of byproducts, such as phosphate. High concentrations of phosphates in the protein synthesis region can promote the phosphorylation of APP. Moreover, β-secretase participate in the subsequent processing of the phosphorylation of APP, which leads to further Aβ accumulation. However, the process in the body that stabilizes the concentration of Aβ can get out of control under certain circumstances. For example, native Aβ can induce the synthesis of additional APP, which undergoes phosphorylation and amyloid processing, leading to increased Aβ concentrations. Meanwhile, high concentrations of Aβ may induce APP synthesis and trigger amyloidosis in peripheral neurons. A portion of Aβ misfolds and accumulates in the brain, forming hydrophobic, extracellular oligomers in the form of plaques and fibers, which negatively affect neurons and synapses (Figure 1) [33].

## 3. Inducing Factors of Amyloidosis in AD

### 3.1. Impaired Autophagy

**Figure 2:**
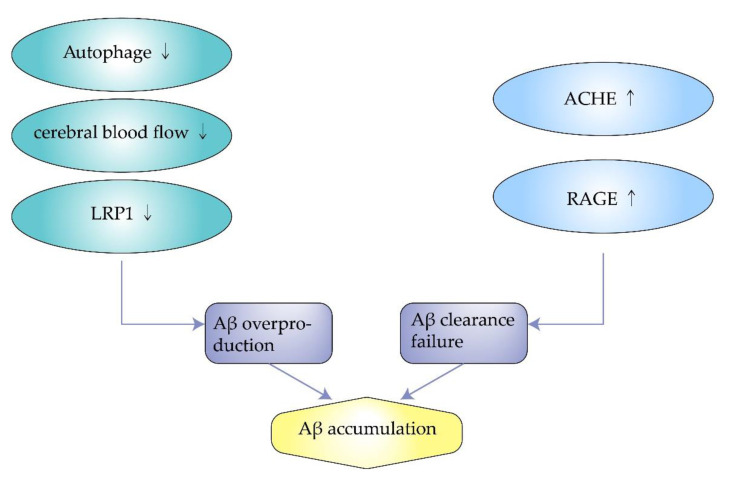
The mechanism of amyloid beta accumulation.

Autophagy engulfs cytoplasmic proteins or dysfunctional organelles, fusing them with lysosomes to form autophagic lysosomes that degrade the contents inside. It allows for the metabolic needs of cells and the renewal of organelles. However, autophagy is downregulated in AD, associated with the metabolism of Aβ. Impaired autophagy is closely related to the pathogenesis of AD because it is associated with the removal of Aβ aggregates and APP. Experiments by F. Zhou et al. showed that proteasome inhibition rapidly reduce APP expression in neuronal cells and facilitate the degradation of APP through autophagy [34]. However, when cellular autophagy is impaired, APP can produce Aβ after a series of reactions. Therefore, modulating the cellular autophagic pathway may be a potential therapeutic approach for Alzheimer’s disease [35,36]. Wu. S et al. found that overexpression of SIRT5 promotes autophagy and reduces the inflammatory response in AD brain and neurons. In contrast, when autophagy is inhibited, the protective effect of SIRT5 on AD neurons is eliminated. Enhanced autophagic degradation pathways protect neurons from neurotoxicity induced by Aβ (Figure 2) [37].

### 3.2. Insufficient Cerebral Blood Flow

Cerebral blood flow (CBF) decrease is a pathological mechanism in Alzheimer’s disease in the early stage. The decline in CBF is mainly due to the contraction of capillaries with contractile pericytes. This process is probably related to oligomeric Aβ [38,39]. Insufficient blood flow may affect the synthesis and clearance of Aβ due to a shortage of energy and oxygen resulting from intracerebral starvation [40]. An effective way to increase cerebral blood flow is exercising more. A tremendous amount of evidence suggests a strong link between physical activity and ameliorating cognitive decline in AD patients. Increasing blood supply to the brain through exercise and a healthy diet may also be effective in preventing Alzheimer’s disease [41,42]. Hashiguchi D et al. found that resistance exercise has positive effects on motor behavior, amyloid loading, and inflammatory pathology in Alzheimer’s disease, providing an idea for improving the clinical symptoms of Alzheimer’s disease [43]. Therefore, the signaling pathways that induce cerebral blood flow redaction may become a target for AD treatment.

### 3.3. Excess Synthesis of ACHE

The central cholinergic system is closely related to mental activity. Acetylcholine (ACh) is the main neurotransmitter whose primary function is to maintain consciousness and plays an important role in learning and memory. One of the pathologies of AD is characterized by a significant decrease in ACh in the brain, leading to the appearance of neurodegenerative symptoms. Moreover, in the synaptic cleft, excessive synthesis of acetylcholinesterase (AChE) degrades acetylcholine resulting in further development of AD. At the same time, amyloid peptides reduce the synthesis of ACh, which is related to the synthesis of Aβ in turn. Low concentrations of Aβ may directly stimulate nicotinic acetylcholine receptors. Dougherty et al. found that 100 pM of Aβ1-42 could activate the α7 isoform of nAchR. Meanwhile, Dineley et al. using X. leavis oocytes found that low concentrations of Aβ were able to inhibit AchR [44,45]. At high concentrations, however, Aβ may reduce the synaptic release of several neurotransmitters while leaving its receptors in a desensitized state. In addition, activation of cholinergic receptors may affect the processing of amyloid precursor proteins, turning them into non-amyloid products and reducing synthesis and aggregation of amyloid. As a result, it reduces neurotoxicity to the brain [46]. Therefore, excessive synthesis of ACHE may indirectly lead to the formation of amyloid, which adversely affects the brain. To date, anti-AChE is still the first-line drug used to reduce the symptoms of AD [47,48].

### 3.4. Decreased Expression of Low-Density Lipoprotein Receptor-Related Protein-1

**Figure 3:**
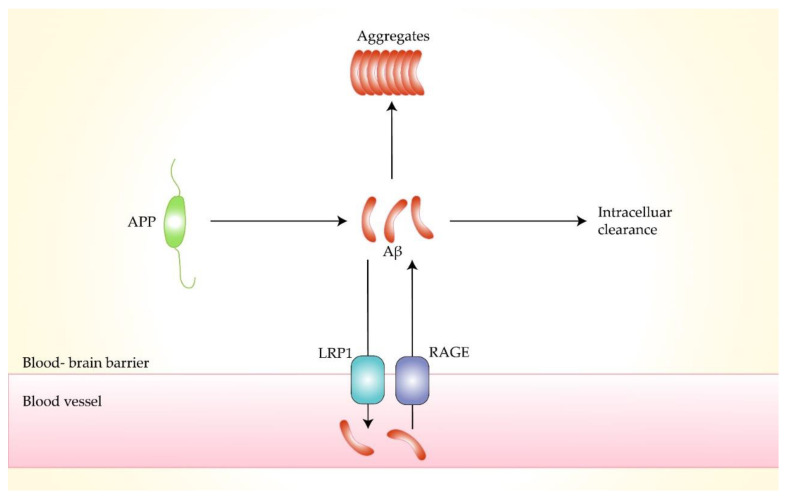
Transportation process of amyloid-beta by low-density lipoprotein receptor-related protein 1 and receptor for advanced glycation end products.

Low-density lipoprotein receptor-related protein 1 (LRP1) is a low-density lipoprotein (LDL) receptor highly expressed in the central nervous system, accelerating the metabolism of amyloid and reducing accumulation. It can bind to amyloid precursor protein (APP) and Aβ, transporting them from the brain into the blood through the blood–brain barrier [49,50,51]. Meanwhile, the endocytosis of LRP1 is related to the up-taking and accumulation of Aβ by lysosomes [52]. Lower expression of LRP1 in capillary endothelial cells and neurons in the brain of AD patients leads to reduced clearance of Aβ, resulting in amyloid accumulation [49]. J. Choid et al. found that in a transgenic mouse model of amyloidosis with knockout the Idol gene, which controls the expression of LRP1—the brain LRP1 was increased, and the amount of soluble and insoluble amyloid was reduced. Amyloid plaque formation decreased, achieving improved neuroinflammation [51]. Patients with high cholesterol are at higher risk of AD than the general population because high cholesterol leads to reduced expression of LRP1 and aggravates the risk of AD [53]. Therefore, LRP1 may be another potential target for the treatment of AD (Figure 3).

### 3.5. Overexpression of Receptor for Advanced Glycosylation End Products

Receptor for advanced glycation end products (RAGE) is a membrane protein that belongs to the immunoglobulin superfamily. It recognizes many types of ligands, such as advanced glycosylation end products (AGEs) and amyloid. Aβ is recognized by RAGE and interacts with RAGE in the cerebral vasculature and finally crosses the blood–brain barrier into the brain [27]. Increased concentration of ligands can lead to upregulation of RAGE expression, which in turn leads to accumulation of toxic proteins in the brain, triggering adverse neurological responses. F. Fang et al. suggested that the RAGE-dependent signaling pathway drives the cleavage of APP by beta- and gamma-secretase to produce Aβ through activation of GSK3beta and p38 MAP kinase [54]. Y.Y. Huang et al. used the RAGE antagonist RP1 to act on an APP/PS1 mouse model of Alzheimer’s disease. The results showed that RP1 reduced the expression of APP and β-secretase, thereby reducing the formation and accumulation of Aβ, improving memory impairment, and reducing amyloid plaque load in the mouse model [55]. Therefore, RAGE is a potential therapeutic target to inhibit abnormal APP metabolism and halt AD progression.

## 4. Pathogenesis of Amyloidosis in AD

### 4.1. Oxidative Damage

The damage caused by excess reactive oxygen species in the body—including phospholipids, proteins, enzymes, and DNA of the cell—is called oxidative damage. Reactive oxygen species (ROS) are an essential factor causing oxidative damage to proteins. The formation of amyloidosis plaques in the brain consisting of aggregated Aβ and metal ions, such as Cu and Fe, is one of the histopathological features of AD. Meanwhile, metal ions coordinating with Aβ and generating complexes are directly involved in ROS production, establishing a relationship between oxidative stress and AD [56]. Moreover, Aβ induces a considerable increase in ROS production, which severely affects neuronal metabolism [57]. High levels of ROS can cause damage to the organism, causing oxidative damage to Aβ peptides and surrounding molecules. At the same time, it can lead to higher ROS accumulation and result in biomolecules damage, forming a vicious circle.

Because it has the highest oxygen use in the body, the brain is particularly sensitive to oxidative damage caused by oxidative stress. Butterfield found that oxidized proteins could be observed in the early stages of AD. Protein and lipid oxidation occurs mainly in Aβ-rich brain regions, while regions with lower Aβ levels do not have high levels of oxidative stress markers [58,59]. Elevated Aβ concentrations were closely associated with protein, lipid, and nucleic acid oxidation in the hippocampus and cortex [60]. Experiments by A. Malkov et al. in 2021 demonstrated that Aβ induces oxidative stress via nox2 and acutely reduces cellular glucose consumption, leading to neural network hyperactivity and behavioral abnormalities in mice [61]. These studies suggest a direct relationship between oxidative damage and Aβ production.

### 4.2. Aberrant Phosphorylation of Tau

The microtubule system is a component of the neural cytoskeleton, which consists of microtubule proteins and microtubule-associated proteins. It is involved in a variety of cellular functions [62]. Among them, tau proteins are the most abundant microtubule-associated proteins. Each tau protein molecule in the brains of Alzheimer’s disease patients contains 5–9 phosphate groups, which are abnormally hyperphosphorylated. Consequently, they cannot promote microtubule assembly formation or maintain microtubule stability. Hurtado et al. found that Aβ-induced activation of intracellular p38MAPK leads to abnormal phosphorylation of tau proteins—accelerating the spatiotemporal progression of tau pathology and increasing the formation of amyloid plaques [63]. In 2020, Wu et al. suggested that Aβ monomer induces phosphorylation of the Ser-214 site of tau protein through the beta2AR-PKA-JNK signaling pathway [64]. Hyperphosphorylated tau leads to impaired microtubule binding and failure to promote microtubule assembly, which leads to microtubule breakdown in late AD. It can also lead to mis-localization of microtubules and an increased propensity to aggregate, causing damage to neurons. However, it is unclear how and whether tau hyperphosphorylation can directly lead to neuronal death [65,66].

### 4.3. Neurofibrillary Tangles

Neurofibrillary tangles (NFTs) are a hallmark pathological change in the cortical cells of AD patients. It is the leading cause of neuronal fiber degeneration and is a hallmark of early brain ageing [67]. NFTs can also be observed in Parkinson’s disease (PD), which shows that it may be the critical factor for neurodegenerative diseases [68]. Hurtado DE et al. used a transgenic (TG) mouse model with both plaque and tangles pathology to explore the interaction between Aβ and NFTs. It showed that Aβ accelerated the formation of NFTs and enhanced tau amyloidosis, especially in the penetrating pathway [63].

### 4.4. Neuronal Damage

The onset and progression of AD are related to neuronal loss and damage. At the same time, Aβ is inextricably linked to neuronal damage. Neurotoxicity due to Aβ aggregation not only impairs neuronal function, but extracellular deposition of plaques can lead to neuronal damage. The occurrence of a series of neuronal damage can cause progressive memory loss and cognitive impairment, resulting in AD development [10]. Studies on the rodent hippocampus have also demonstrated that Aβ oligomers separated from the cerebral cortex of AD patients and their tissue cultures decreased, significantly inhibited long-time course enhancement, disrupted synaptic plasticity, and led to impairment of memory function [69,70]. The binding of Aβ oligomers to neuronal membranes induces the formation of membrane perforations, causing an inward flow of calcium ions and increasing the release of calcium ions, leading to a delayed synaptic failure generated through vesicles depletion [71,72,73]. Furthermore, Aβ accumulation cause alterations in synaptic mitochondria. Aβ also induces phosphorylation of microtubule-associated protein tau through specific kinase activation, leading to mitochondrial toxicity in neurons [74].

## 5. Therapeutic Directions of Amyloidosis in AD

Although the global life expectancy is increasing year by year, the number of people with Alzheimer’s disease is increasing. Alzheimer’s disease cannot be cured completely, and the available medications can only relieve AD symptoms. There are some drugs used in clinical treatment in ID. However, these drugs are individually variable, and some users also experience many adverse drug reactions and side effects. Therefore, there is a strong need to investigate effective treatments for AD. Multiple pathways involved in the etiology of AD can be targeted simultaneously. Therefore, the development of non-toxic and stable inhibitors of amyloid synthesis is a significant challenge. Extensive research has been conducted on targeted drugs for Aβ.

### 5.1. Inhibition of Aβ Production

A 2019 study used transgenic mice that overexpressed APP and gave them the BACE1 inhibitor NB-360. Aβ in the cerebrospinal fluid and brain tissue of the mice significantly reduced after treatment. This study suggests that NB-360 has an inhibitory effect on Aβ production in vivo [75]. Baranger K et al. investigated the role of RS 67333, a partial agonist of 5HT4R, in 5xFAD, in a mouse model of AD. They found that after four months of the administration, the mice showed a reduction in pathology stigmata. Their learning and memory significantly improved. It suggested that 5-HT4R agonists can inhibit the production of Aβ and thus improve AD symptoms [76].

### 5.2. Reduction in Aβ Deposition

The toxic accumulation of Aβ in the brain is one of the pathological manifestations of AD, so reducing the deposition of Aβ is an important target for drugs. Du, Y et al. in 2016 found that APP/PS1 mice treated with kynylpinoside showed a significant reduction in the number of plaques in the cerebral cortex and hippocampus, while Aβ accumulation in the cerebral cortex and hippocampus remained high in the control group. This suggests that kynylpinoside can reduce Aβ accumulation in the brain, providing an excellent therapeutic idea [77]. Zhao C et al. found that kynepin glycosides led to reduced Aβ accumulation by inhibiting the excessive activation of MAPK signaling mediated by Aβ-RAGE interactions [78]. Moreover, scFv-h3D6, an anti-Aβ single-chain variable fragment which is derived from the antibody bapineuzumab, has a similar effect in AD [79]. In addition, herbal medicine is an effective therapy for Alzheimer’s disease in the reduction in Aβ deposition. Pharmaceutical treatments using natural compounds derived from plants are economical and result in relatively few side effects. Yang C. et al. studied the effects of Paeonia alba on amyloidosis and neuronal degeneration in APP/PS1 model mice. They found that LDL receptor-related protein-1 levels were upregulated, and late glycosylation end-product receptor deposition was downregulated in the brains of mice treated with Paeonia. Moreover, this was accompanied by a reduction in Aβ deposition and cognitive impairment, releasing the neuronal damage [80] It has been reported that Angelica Sinensis Shao Yao San reduced Aβ41-42 deposition in the brain of APP/PS1 mice and improved amyloidosis and neuronal degeneration in Alzheimer’s disease through a similar molecular mechanism [80]. Berberine promotes the restoration of cerebral blood flow by promoting the integrity of neovascular structures and improving vascular function in the brain. It also reduces the accumulation of Aβ to improve cognition [81].

### 5.3. Protection of Neurons

Neuronal damage and loss are significant causes of cognitive dysfunction in AD, which is tightly associated with the accumulation of Aβ in the brain. Therefore, preserving existing synapses and preventing neuronal loss is an important strategy to protect cognitive function in AD patients. Maiti. P. et al. used the 5xFAD mouse model and age-matched wild-type mice, divided them into two groups, and orally administered solid lipid curcumin pellets (SLCP) and equal amounts of fugitive agents. After two months, it was evaluated that SLCP protected these nerve injuries to some extent and preserved the typical morphology of dendrites. This experiment suggests that SLCP protects neurons and improves cognitive function in 5xFAD mice [82]. A 2020 study indicated that Shenzao Jiannao oral liquid (SZJN), a traditional Chinese preparation, could improve cognitive function by preventing neuronal death and triggering endogenous neurogenesis. Therefore, SZJN may be considered a promising drug to restore neuronal damage and stop the deterioration of AD in patients [83].

## 6. Summary and Perspectives

There is substantial evidence to support the mechanism by which amyloid accumulation in the brain increases the risk of Alzheimer’s disease. Amyloid accumulation is caused by many factors, including impairment of cellular autophagy, low cerebral blood flow, excessive synthesis of ACHE, downregulation of LDL-related protein-1 expression, and overexpression of receptors for advanced glycosylation end products. On the other hand, studies on Alzheimer’s disease have shown that oxidative damage, tau protein hyperphosphorylation, and neurofibrillary tangles are among the etiologies of amyloid-related AD. Furthermore, in recent years, drugs targeting the accumulation of amyloid have shown potential for future treatment. Overall, based on plausible in vivo and in vitro studies, it is reasonable to assume that Aβ is an important drug target for the treatment of AD. Further studies should focus on the molecular mechanisms of amyloid and related therapeutics.

## Data Availability

Not applicable.

## References

[1] Long J.M., Holtzman D.M. (2019). Alzheimer Disease: An Update on Pathobiology and Treatment Strategies. Cell.

[2] Prince M.J., Wimo A., Guerchet M.M., Ali G.C., Wu Y.-T., Prina M. (2015). World Alzheimer Report 2015—The Global Impact of Dementia: An Analysis of Prevalence, Incidence, Cost and Trends.

[3] Liu Y.S., Wang Y.M., Zha D.J. (2021). Brain Functional and Structural Changes in Alzheimer’s Disease With Sleep Disorders: A Systematic Review. Front. Psychiatry.

[4] Hohenfeld C., Kuhn H., Muller C., Nellessen N., Ketteler S., Heinecke A., Goebel R., Shah N.J., Schulz J.B., Reske M. (2020). Changes in brain activation related to visuo-spatial memory after real-time fMRI neurofeedback training in healthy elderly and Alzheimer’s disease. Behav. Brain Res..

[5] Brookmeyer R., Johnson E., Ziegler-Graham K., Arrighi H.M. (2007). Forecasting the global burden of Alzheimer’s disease. Alzheimers Dement..

[6] Arvanitakis Z., Shah R.C., Bennett D.A. (2019). Diagnosis and Management of Dementia: Review. JAMA.

[7] Alzheimer’s A. (2016). 2016 Alzheimer’s disease facts and figures. Alzheimers Dement..

[8] Vasefi M., Ghaboolian-Zare E., Abedelwahab H., Osu A. (2020). Environmental toxins and Alzheimer’s disease progression. Neurochem. Int..

[9] Zhu J.B., Tan C.C., Tan L., Yu J.T. (2017). State of Play in Alzheimer’s Disease Genetics. J. Alzheimers Dis..

[10] Prasansuklab A., Tencomnao T. (2013). Amyloidosis in Alzheimer’s Disease: The Toxicity of Amyloid Beta (Aβ), Mechanisms of Its Accumulation and Implications of Medicinal Plants for Therapy. Evid. Based Complement. Alternat. Med..

[11] Selkoe D.J., Hardy J. (2016). The amyloid hypothesis of Alzheimer’s disease at 25 years. EMBO Mol. Med..

[12] Hillen H. (2019). The Beta Amyloid Dysfunction (BAD) Hypothesis for Alzheimer’s Disease. Front. Neurosci..

[13] Forloni G., Artuso V., La Vitola P., Balducci C. (2016). Oligomeropathies and pathogenesis of Alzheimer and Parkinson’s diseases. Mov. Disord..

[14] Westermark P., Benson M.D., Buxbaum J.N., Cohen A.S., Frangione B., Ikeda S., Masters C.L., Merlini G., Saraiva M.J., Sipe J.D. (2007). A primer of amyloid nomenclature. Amyloid.

[15] Glenner G.G., Wong C.W. (2012). Alzheimer’s disease: Initial report of the purification and characterization of a novel cerebrovascular amyloid protein. 1984. Biochem. Biophys. Res. Commun..

[16] Merlini G., Bellotti V. (2003). Molecular mechanisms of amyloidosis. N. Engl. J. Med..

[17] Rice H.C., de Malmazet D., Schreurs A., Frere S., Van Molle I., Volkov A.N., Creemers E., Vertkin I., Nys J., Ranaivoson F.M. (2019). Secreted amyloid-beta precursor protein functions as a GABABR1a ligand to modulate synaptic transmission. Science.

[18] O’Brien R.J., Wong P.C. (2011). Amyloid precursor protein processing and Alzheimer’s disease. Annu. Rev. Neurosci..

[19] Thinakaran G., Koo E.H. (2008). Amyloid precursor protein trafficking, processing, and function. J. Biol. Chem..

[20] Blennow K., de Leon M.J., Zetterberg H. (2006). Alzheimer’s disease. Lancet.

[21] Sadleir K.R., Kandalepas P.C., Buggia-Prevot V., Nicholson D.A., Thinakaran G., Vassar R. (2016). Presynaptic dystrophic neurites surrounding amyloid plaques are sites of microtubule disruption, BACE1 elevation, and increased Abeta generation in Alzheimer’s disease. Acta Neuropathol..

[22] Nalivaeva N.N., Turner A.J. (2013). The amyloid precursor protein: A biochemical enigma in brain development, function and disease. FEBS Lett..

[23] Storey E., Cappai R. (1999). The amyloid precursor protein of Alzheimer’s disease and the Abeta peptide. Neuropathol. Appl. Neurobiol..

[24] Rogaev E.I. (1999). Genetic factors and a polygenic model of Alzheimer’s disease. Genetika.

[25] Devkota S., Williams T.D., Wolfe M.S. (2021). Familial Alzheimer’s disease mutations in amyloid protein precursor alter proteolysis by gamma-secretase to increase amyloid beta-peptides of >/=45 residues. J. Biol. Chem..

[26] Selkoe D.J. (2001). Clearing the brain’s amyloid cobwebs. Neuron.

[27] Deane R., Du Yan S., Submamaryan R.K., LaRue B., Jovanovic S., Hogg E., Welch D., Manness L., Lin C., Yu J. (2003). RAGE mediates amyloid-beta peptide transport across the blood-brain barrier and accumulation in brain. Nat. Med..

[28] Deane R., Bell R.D., Sagare A., Zlokovic B.V. (2009). Clearance of amyloid-beta peptide across the blood-brain barrier: Implication for therapies in Alzheimer’s disease. CNS Neurol. Disord. Drug Targets.

[29] Levin O.S., Vasenina E.E. (2016). Twenty-five years of the amyloid hypothesis of alzheimer disease: Advances, failures and new perspectives. Zh. Nevrol. Psikhiatr. Im. SS Korsakova.

[30] Jack C.R., Bennett D.A., Blennow K., Carrillo M.C., Dunn B., Haeberlein S.B., Holtzman D.M., Jagust W., Jessen F., Karlawish J. (2018). NIA-AA Research Framework: Toward a biological definition of Alzheimer’s disease. Alzheimers Dement..

[31] Fukumoto H., Tomita T., Matsunaga H., Ishibashi Y., Saido T.C., Iwatsubo T. (1999). Primary cultures of neuronal and non-neuronal rat brain cells secrete similar proportions of amyloid beta peptides ending at A beta40 and A beta42. Neuroreport.

[32] Tsitsopoulos P.P., Marklund N. (2013). Amyloid-beta Peptides and Tau Protein as Biomarkers in Cerebrospinal and Interstitial Fluid Following Traumatic Brain Injury: A Review of Experimental and Clinical Studies. Front. Neurol..

[33] Maltsev A.V., Santockyte R., Bystryak S., Galzitskaya O.V. (2014). Activation of neuronal defense mechanisms in response to pathogenic factors triggering induction of amyloidosis in Alzheimer’s disease. J. Alzheimers Dis..

[34] Zhou F., van Laar T., Huang H., Zhang L. (2011). APP and APLP1 are degraded through autophagy in response to proteasome inhibition in neuronal cells. Protein. Cell.

[35] Nixon R.A. (2007). Autophagy, amyloidogenesis and Alzheimer disease. J. Cell Sci..

[36] Hung S.Y., Huang W.P., Liou H.C., Fu W.M. (2009). Autophagy protects neuron from Abeta-induced cytotoxicity. Autophagy.

[37] Wu S., Wei Y., Li J., Bai Y., Yin P., Wang S. (2021). SIRT5 Represses Neurotrophic Pathways and Abeta Production in Alzheimer’s Disease by Targeting Autophagy. ACS Chem. Neurosci..

[38] Li D., Liu Y., Zeng X., Xiong Z., Yao Y., Liang D., Qu H., Xiang H., Yang Z., Nie L. (2020). Quantitative Study of the Changes in Cerebral Blood Flow and Iron Deposition During Progression of Alzheimer’s Disease. J. Alzheimers Dis..

[39] Fazlollahi A., Calamante F., Liang X., Bourgeat P., Raniga P., Dore V., Fripp J., Ames D., Masters C.L., Rowe C.C. (2020). Increased cerebral blood flow with increased amyloid burden in the preclinical phase of alzheimer’s disease. J. Magn. Reson. Imaging.

[40] Winchester J., Dick M.B., Gillen D., Reed B., Miller B., Tinklenberg J., Mungas D., Chui H., Galasko D., Hewett L. (2013). Walking stabilizes cognitive functioning in Alzheimer’s disease (AD) across one year. Arch. Gerontol. Geriatr..

[41] Maesako M., Uemura K., Kubota M., Kuzuya A., Sasaki K., Hayashida N., Asada-Utsugi M., Watanabe K., Uemura M., Kihara T. (2012). Exercise is more effective than diet control in preventing high fat diet-induced beta-amyloid deposition and memory deficit in amyloid precursor protein transgenic mice. J. Biol. Chem..

[42] Intlekofer K.A., Cotman C.W. (2013). Exercise counteracts declining hippocampal function in aging and Alzheimer’s disease. Neurobiol. Dis..

[43] Hashiguchi D., Campos H.C., Wuo-Silva R., Faber J., Gomes da Silva S., Coppi A.A., Arida R.M., Longo B.M. (2020). Resistance Exercise Decreases Amyloid Load and Modulates Inflammatory Responses in the APP/PS1 Mouse Model for Alzheimer’s Disease. J. Alzheimers Dis..

[44] Dougherty J.J., Wu J., Nichols R.A. (2003). Beta-amyloid regulation of presynaptic nicotinic receptors in rat hippocampus and neocortex. J. Neurosci..

[45] Dineley K.T., Bell K.A., Bui D., Sweatt J.D. (2002). beta-Amyloid peptide activates alpha 7 nicotinic acetylcholine receptors expressed in Xenopus oocytes. J. Biol. Chem..

[46] Govoni S., Mura E., Preda S., Racchi M., Lanni C., Grilli M., Zappettini S., Salamone A., Olivero G., Pittaluga A. (2014). Dangerous liaisons between beta-amyloid and cholinergic neurotransmission. Curr. Pharm. Des..

[47] Marco-Contelles J., Unzeta M., Bolea I., Esteban G., Ramsay R.R., Romero A., Martinez-Murillo R., Carreiras M.C., Ismaili L. (2016). ASS234, As a New Multi-Target Directed Propargylamine for Alzheimer’s Disease Therapy. Front. Neurosci..

[48] Simoni E., Bartolini M., Abu I.F., Blockley A., Gotti C., Bottegoni G., Caporaso R., Bergamini C., Andrisano V., Cavalli A. (2017). Multitarget drug design strategy in Alzheimer’s disease: Focus on cholinergic transmission and amyloid-beta aggregation. Future Med. Chem..

[49] Donahue J.E., Flaherty S.L., Johanson C.E., Duncan J.A., Silverberg G.D., Miller M.C., Tavares R., Yang W., Wu Q., Sabo E. (2006). RAGE, LRP-1, and amyloid-beta protein in Alzheimer’s disease. Acta Neuropathol..

[50] Sagare A., Deane R., Bell R.D., Johnson B., Hamm K., Pendu R., Marky A., Lenting P.J., Wu Z., Zarcone T. (2007). Clearance of amyloid-beta by circulating lipoprotein receptors. Nat. Med..

[51] Choi J., Gao J., Kim J., Hong C., Kim J., Tontonoz P. (2015). The E3 ubiquitin ligase Idol controls brain LDL receptor expression, ApoE clearance, and Abeta amyloidosis. Sci. Transl. Med..

[52] Fuentealba R.A., Liu Q., Zhang J., Kanekiyo T., Hu X., Lee J.M., LaDu M.J., Bu G. (2010). Low-density lipoprotein receptor-related protein 1 (LRP1) mediates neuronal Abeta42 uptake and lysosomal trafficking. PLoS ONE.

[53] Zhou R., Chen L.L., Yang H., Li L., Liu J., Chen L., Hong W.J., Wang C.G., Ma J.J., Huang J. (2021). Effect of High Cholesterol Regulation of LRP1 and RAGE on Abeta Transport Across the Blood-Brain Barrier in Alzheimer’s Disease. Curr. Alzheimer Res..

[54] Fang F., Yu Q., Arancio O., Chen D., Gore S.S., Yan S.S., Yan S.F. (2018). RAGE mediates Abeta accumulation in a mouse model of Alzheimer’s disease via modulation of beta- and gamma-secretase activity. Hum. Mol. Genet..

[55] Huang Y.Y., Fang N., Luo H.R., Gao F., Zou Y., Zhou L.L., Zeng Q.P., Fang S.S., Xiao F., Zheng Q. (2020). RP1, a RAGE antagonist peptide, can improve memory impairment and reduce Abeta plaque load in the APP/PS1 mouse model of Alzheimer’s disease. Neuropharmacology.

[56] Halliwell B. (2006). Oxidative stress and neurodegeneration: Where are we now?. J. Neurochem..

[57] Sotolongo K., Ghiso J., Rostagno A. (2020). Nrf2 activation through the PI3K/GSK-3 axis protects neuronal cells from Abeta-mediated oxidative and metabolic damage. Alzheimers Res. Ther..

[58] Butterfield D.A. (2014). The 2013 SFRBM discovery award: Selected discoveries from the butterfield laboratory of oxidative stress and its sequela in brain in cognitive disorders exemplified by Alzheimer disease and chemotherapy induced cognitive impairment. Free Radic. Biol. Med..

[59] Butterfield D.A., Bader Lange M.L., Sultana R. (2010). Involvements of the lipid peroxidation product, HNE, in the pathogenesis and progression of Alzheimer’s disease. Biochim. Biophys. Acta.

[60] Butterfield D.A., Lauderback C.M. (2002). Lipid peroxidation and protein oxidation in Alzheimer’s disease brain: Potential causes and consequences involving amyloid beta-peptide-associated free radical oxidative stress. Free Radic. Biol. Med..

[61] Malkov A., Popova I., Ivanov A., Jang S.S., Yoon S.Y., Osypov A., Huang Y., Zilberter Y., Zilberter M. (2021). Abeta initiates brain hypometabolism, network dysfunction and behavioral abnormalities via NOX2-induced oxidative stress in mice. Commun. Biol..

[62] Kapasi A., Leurgans S.E., Arvanitakis Z., Barnes L.L., Bennett D.A., Schneider J.A. (2021). Abeta (Amyloid Beta) and Tau Tangle Pathology Modifies the Association Between Small Vessel Disease and Cortical Microinfarcts. Stroke.

[63] Hurtado D.E., Molina-Porcel L., Iba M., Aboagye A.K., Paul S.M., Trojanowski J.Q., Lee V.M. (2010). A{beta} accelerates the spatiotemporal progression of tau pathology and augments tau amyloidosis in an Alzheimer mouse model. Am. J. Pathol..

[64] Wu H., Wei S., Huang Y., Chen L., Wang Y., Wu X., Zhang Z., Pei Y., Wang D. (2020). Abeta monomer induces phosphorylation of Tau at Ser-214 through beta2AR-PKA-JNK signaling pathway. FASEB J..

[65] Gilley J., Ando K., Seereeram A., Rodriguez-Martin T., Pooler A.M., Sturdee L., Anderton B.H., Brion J.P., Hanger D.P., Coleman M.P. (2016). Mislocalization of neuronal tau in the absence of tangle pathology in phosphomutant tau knockin mice. Neurobiol. Aging.

[66] Xia Y., Prokop S., Giasson B.I. (2021). “Don’t Phos Over Tau”: Recent developments in clinical biomarkers and therapies targeting tau phosphorylation in Alzheimer’s disease and other tauopathies. Mol. Neurodegener..

[67] Minati L., Edginton T., Bruzzone M.G., Giaccone G. (2009). Current concepts in Alzheimer’s disease: A multidisciplinary review. Am. J. Alzheimers Dis. Other Demen..

[68] Goedert M. (2015). Neurodegeneration. Alzheimer’s and Parkinson’s diseases: The prion concept in relation to assembled Abeta, tau, and alpha-synuclein. Science.

[69] Walsh D.M., Klyubin I., Fadeeva J.V., Cullen W.K., Anwyl R., Wolfe M.S., Rowan M.J., Selkoe D.J. (2002). Naturally secreted oligomers of amyloid beta protein potently inhibit hippocampal long-term potentiation in vivo. Nature.

[70] Shankar G.M., Li S., Mehta T.H., Garcia-Munoz A., Shepardson N.E., Smith I., Brett F.M., Farrell M.A., Rowan M.J., Lemere C.A. (2008). Amyloid-beta protein dimers isolated directly from Alzheimer’s brains impair synaptic plasticity and memory. Nat. Med..

[71] Parodi J., Sepulveda F.J., Roa J., Opazo C., Inestrosa N.C., Aguayo L.G. (2010). Beta-amyloid causes depletion of synaptic vesicles leading to neurotransmission failure. J. Biol. Chem..

[72] Sepulveda F.J., Parodi J., Peoples R.W., Opazo C., Aguayo L.G. (2010). Synaptotoxicity of Alzheimer beta amyloid can be explained by its membrane perforating property. PLoS ONE.

[73] Peters C., Fernandez-Perez E.J., Burgos C.F., Espinoza M.P., Castillo C., Urrutia J.C., Streltsov V.A., Opazo C., Aguayo L.G. (2013). Inhibition of amyloid beta-induced synaptotoxicity by a pentapeptide derived from the glycine zipper region of the neurotoxic peptide. Neurobiol. Aging.

[74] Du H., Guo L., Yan S.S. (2012). Synaptic mitochondrial pathology in Alzheimer’s disease. Antioxid. Redox Signal..

[75] Schelle J., Wegenast-Braun B.M., Fritschi S.K., Kaeser S.A., Jährling N., Eicke D., Skodras A., Beschorner N., Obermueller U., Häsler L.M. (2019). Early Aβ reduction prevents progression of cerebral amyloid angiopathy. Ann. Neurol..

[76] Baranger K., Giannoni P., Girard S.D., Girot S., Gaven F., Stephan D., Migliorati M., Khrestchatisky M., Bockaert J., Marchetti-Gauthier E. (2017). Chronic treatments with a 5-HT(4) receptor agonist decrease amyloid pathology in the entorhinal cortex and learning and memory deficits in the 5xFAD mouse model of Alzheimer’s disease. Neuropharmacology.

[77] Du Y., Qu J., Zhang W., Bai M., Zhou Q., Zhang Z., Li Z., Miao J. (2016). Morin reverses neuropathological and cognitive impairments in APPswe/PS1dE9 mice by targeting multiple pathogenic mechanisms. Neuropharmacology.

[78] Zhao C., Zhang H., Li H., Lv C., Liu X., Li Z., Xin W., Wang Y., Zhang W. (2017). Geniposide ameliorates cognitive deficits by attenuating the cholinergic defect and amyloidosis in middle-aged Alzheimer model mice. Neuropharmacology.

[79] Esquerda-Canals G., Roda A.R., Marti-Clua J., Montoliu-Gaya L., Rivera-Hernandez G., Villegas S. (2019). Treatment with scFv-h3D6 Prevented Neuronal Loss and Improved Spatial Memory in Young 3xTg-AD Mice by Reducing the Intracellular Amyloid-beta Burden. J. Alzheimers Dis..

[80] Yang C., Mo Y.S., Chen H.F., Huang Y.H., Li S.L., Wang H., Huang S.Q., Chang X., Du Q., Wang Q. (2021). The effects of Danggui-Shaoyao-San on neuronal degeneration and amyloidosis in mouse and its molecular mechanism for the treatment of Alzheimer’s disease. J. Integr. Neurosci..

[81] Chen M., Li L., Liu C., Song L. (2020). Berberine attenuates Abeta-induced neuronal damage through regulating miR-188/NOS1 in Alzheimer’s disease. Mol. Cell Biochem..

[82] Maiti P., Bowers Z., Bourcier-Schultz A., Morse J., Dunbar G.L. (2021). Preservation of dendritic spine morphology and postsynaptic signaling markers after treatment with solid lipid curcumin particles in the 5xFAD mouse model of Alzheimer’s amyloidosis. Alzheimers Res. Ther..

[83] Xiao H., Li H., Song H., Kong L., Yan X., Li Y., Deng Y., Tai H., Wu Y., Ni Y. (2020). Shenzao jiannao oral liquid, an herbal formula, ameliorates cognitive impairments by rescuing neuronal death and triggering endogenous neurogenesis in AD-like mice induced by a combination of Abeta42 and scopolamine. J. Ethnopharmacol..

